# Endogenous reparative pluripotent Muse cells with a unique immune privilege system: Hint at a new strategy for controlling acute and chronic inflammation

**DOI:** 10.3389/fphar.2022.1027961

**Published:** 2022-10-19

**Authors:** Yasumasa Kuroda, Yo Oguma, Kerrigan Hall, Mari Dezawa

**Affiliations:** Department of Stem Cell Biology and Histology, Tohoku University Graduate School of Medicine, Sendai, Japan

**Keywords:** reparative stem cells, pluripotent stem cells, cell therapy, anti-inflammatory, anti-fibrotic, tissue repair, stress tolerant

## Abstract

Multilineage-differentiating stress enduring (Muse) cells, non-tumorigenic endogenous pluripotent stem cells, reside in the bone marrow (BM), peripheral blood, and connective tissue as pluripotent surface marker SSEA-3(+) cells. They express other pluripotent markers, including Nanog, Oct3/4, and Sox2 at moderate levels, differentiate into triploblastic lineages, self-renew at a single cell level, and exhibit anti-inflammatory effects. Cultured mesenchymal stromal cells (MSCs) and fibroblasts contain several percent of SSEA-3(+)-Muse cells. Circulating Muse cells, either endogenous or administered exogenously, selectively accumulate at the damaged site by sensing sphingosine-1-phosphate (S1P), a key mediator of inflammation, produced by damaged cells and replace apoptotic and damaged cells by spontaneously differentiating into multiple cells types that comprise the tissue and repair the tissue. Thus, intravenous injection is the main route for Muse cell treatment, and surgical operation is not necessary. Furthermore, gene introduction or cytokine induction are not required for generating pluripotent or differentiated states prior to treatment. Notably, allogenic and xenogenic Muse cells escape host immune rejection after intravenous injection and survive in the tissue as functioning cells over 6 and ∼2 months, respectively, without immunosuppressant treatment. Since Muse cells survive in the host tissue for extended periods of time, therefore their anti-inflammatory, anti-fibrotic, and trophic effects are long-lasting. These unique characteristics have led to the administration of Muse cells *via* intravenous drip in clinical trials for stroke, acute myocardial infarction, epidermolysis bullosa, spinal cord injury, neonatal hypoxic ischemic encephalopathy, amyotrophic lateral sclerosis, and COVID-19 acute respiratory distress syndrome without HLA-matching or immunosuppressive treatment.

## Introduction

Excessive and prolonged inflammation leads to tissue destruction and fibrosis, eventually leading to organ dysfunction (Wynn and Ramalingam, 2012). Currently, administering drugs that suppress inflammatory mediators is the primary method for controlling inflammation (Kulmatycki and Jamali, 2005). Recently, however, the use of cell therapy for anti-inflammatory treatment has gained attraction for clinical applications (Saeedi et al., 2019). Unlike drugs, autologous cells are not quickly metabolized, nor are they rapidly eliminated from the body, but remain in the body or tissues where inflammatory destruction has occurred for a certain period of time. Therefore, cell therapy is expected to be a new approach to effectively control inflammation.

However, the clinical applicability of autologous cells is actually rather limited. Cells cannot be collected if the patient’s condition is poor. Furthermore, cell collection may be difficult depending on the patient’s age. With these issues in mind, donor-derived allogenic cell therapy poses as a more practical option. In the case of allogenic cell therapy, however, human leukocyte antigen (HLA) matching is strictly required to avoid immune rejection. Even if cells possess the power to control inflammation, they serve no use if they are ultimately rejected. For these reasons, mesenchymal stromal cells (MSCs) are currently being applied to diseases such as graft-versus-host disease (GVHD) since MSCs have immunomodulatory and immunosuppressive effects (Hori et al., 2016; Tanaka et al., 2018). Even for allogenic MSCs, however, immunologic rejection has been reported (Tanaka et al., 2018).

**FIGURE 1:**
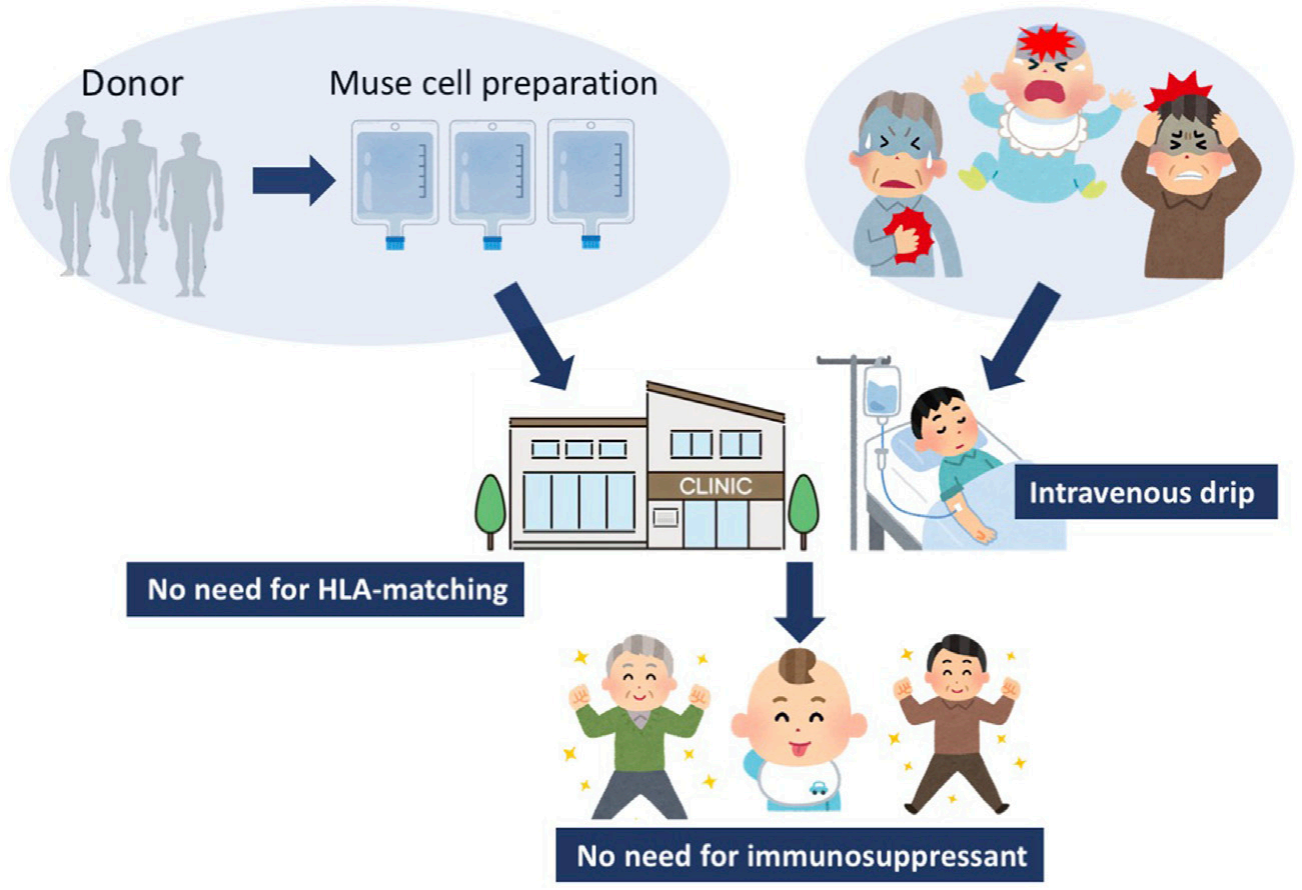
Strategy for Muse cell treatment. Muse cells, collectable from donor sources such as the BM, adipose tissue, and umbilical cord, are expanded to produce Muse cell preparations, and directly delivered to patients by intravenous drip without HLA-matching test or immunosuppressant treatment. Since Muse cells are pluripotent-like, they might be able to target various kinds of diseases.

**FIGURE 2:**
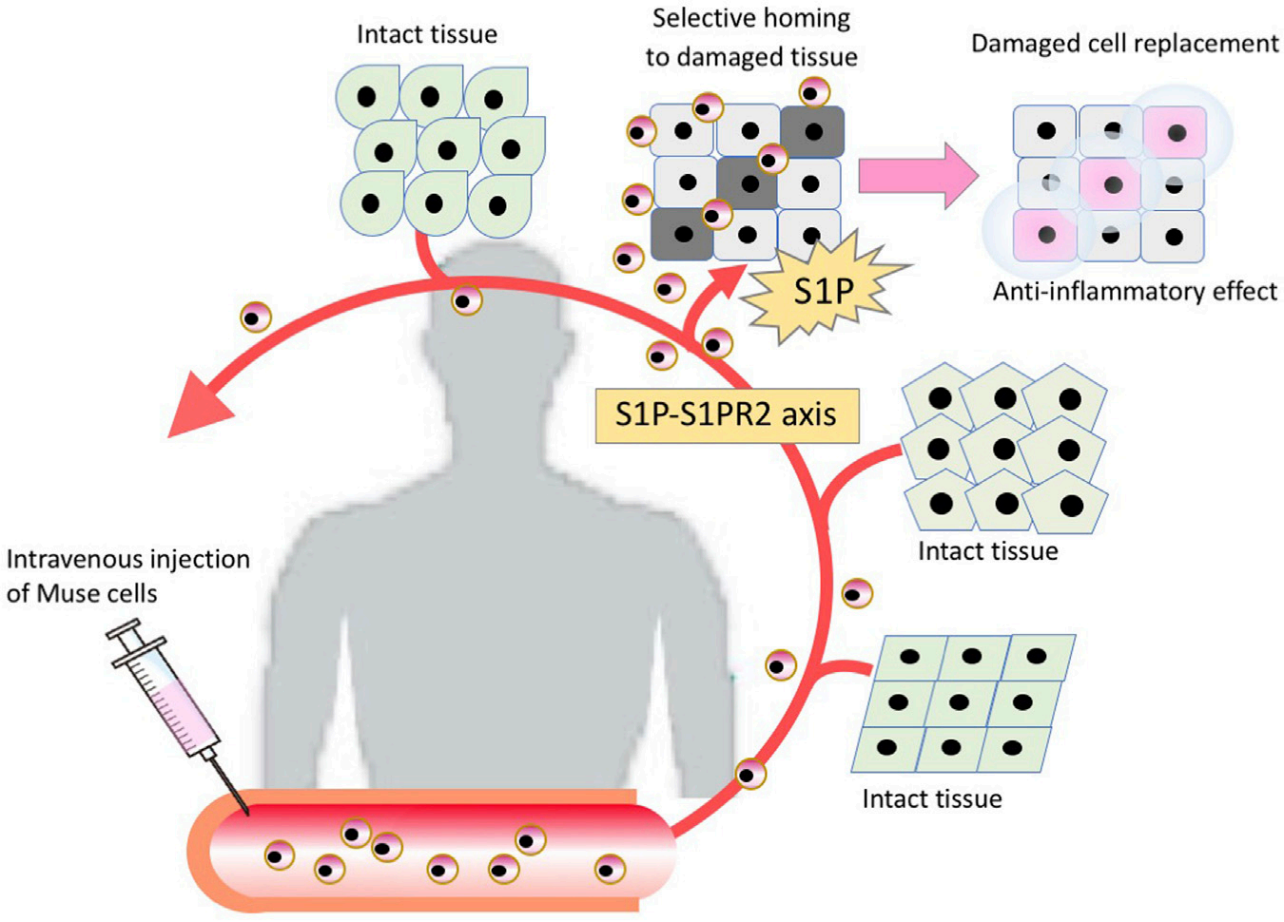
The mechanism of tissue repair by intravenously injected Muse cells. Intravenously injected Muse cells sense the damaged site by the S1P-S1PR2 axis, home to the damaged site and replace damaged cells by spontaneous differentiation into tissue-appropriate cells to repair the tissue.

Multilineage-differentiating stress enduring (Muse) cells, endogenous pluripotent-like reparative stem cells, possess unique characteristics that are not seen in other stem cells, including MSCs, and have been applied to clinical trials (Kuroda et al., 2010; Noda et al., 2020; Fujita et al., 2021). A beneficial feature of donor-derived allogenic Muse cells is that they can be directly administered to patients without HLA matching or immunosuppressive therapy (Figure 1) (Yamada et al., 2018). Intravenously infused donor-derived Muse cells are able to selectively accumulate at the damage site by sharply sensing the alert signal sphingosine-1-phosphate (S1P), a key mediator of inflammation, instead of getting trapped in the lung capillaries (Figure 2) (Yamada et al., 2018). At the damage site, they can deliver anti-inflammatory, anti-apoptotic, anti-fibrosis, and tissue-protective effects and repair the tissue by replacing damaged or apoptotic cells by spontaneous differentiation into tissue-constituent cells (Figure 2) (Uchida et al., 2017b; Yabuki et al., 2018; Yamada et al., 2018; Shono et al., 2021). If we handle Muse cells adequately by understanding their properties, they may have great potential to effectively control not only acute but also chronic inflammation.

## Unique characters of Muse cells

### Muse cells are endogenous to the body

**FIGURE 3:**
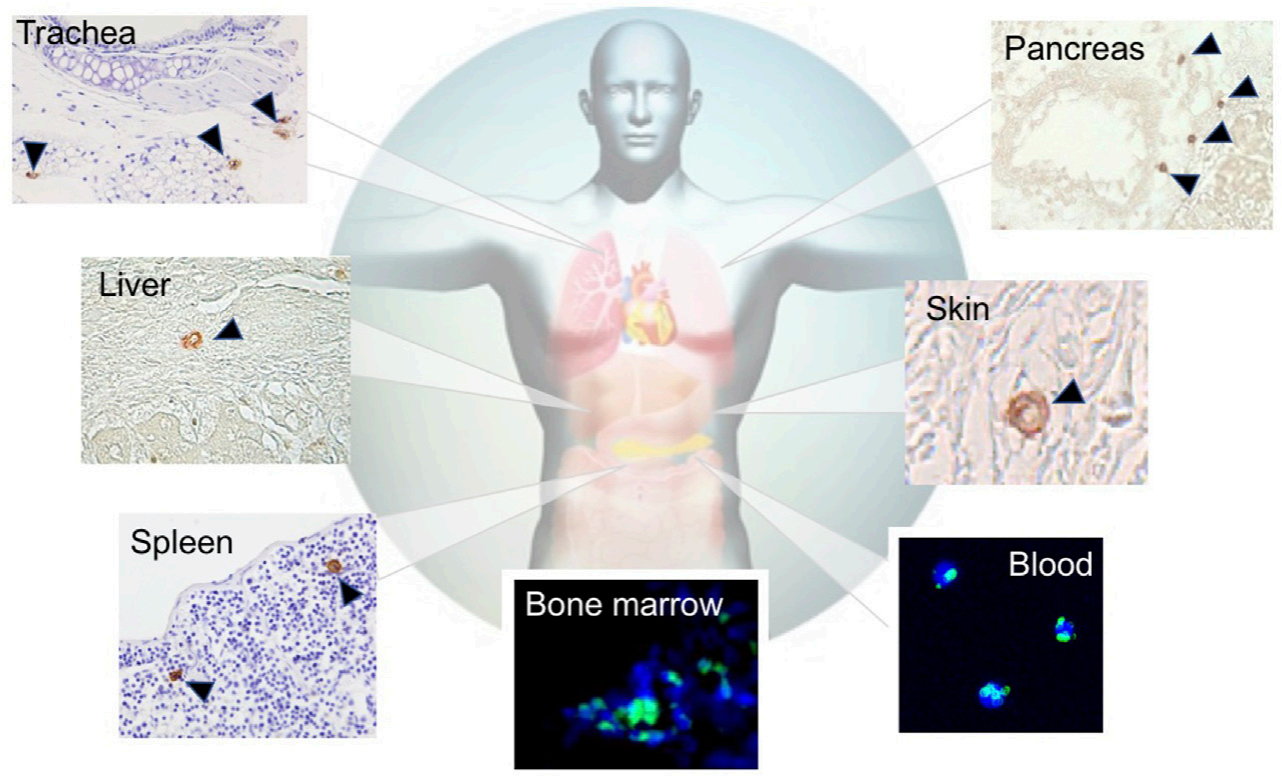
Distribution of endogenous Muse cells *in vivo*. Muse cells, identified as SSEA-3(+), locate in the BM (green signal) [(Hori et al., 2016)], peripheral blood (green signal) (Sato et al., 2020), and connective tissue of various organs (brown signal) (Dezawa, 2016).

Muse cells are endogenous pluripotent-like stem cells that exhibit differentiation into ectodermal, endodermal, and mesodermal lineages, self-renewability, stress tolerance, and reparative function *in vivo* (Kuroda et al., 2010; Wakao et al., 2011; Alessio et al., 2017, 2018). They are identified as pluripotent surface marker stage-specific embryonic antigen 3 positive, SSEA-3(+), in various tissues such as the bone marrow (BM), peripheral blood, and connective tissues. They exhibit low telomerase activity and do not form teratomas after transplantation *in vivo* (Figure 3) (Kuroda et al., 2010; Wakao et al., 2011; Heneidi et al., 2013; Alessio et al., 2017; Gimeno et al., 2017).

**FIGURE 4:**
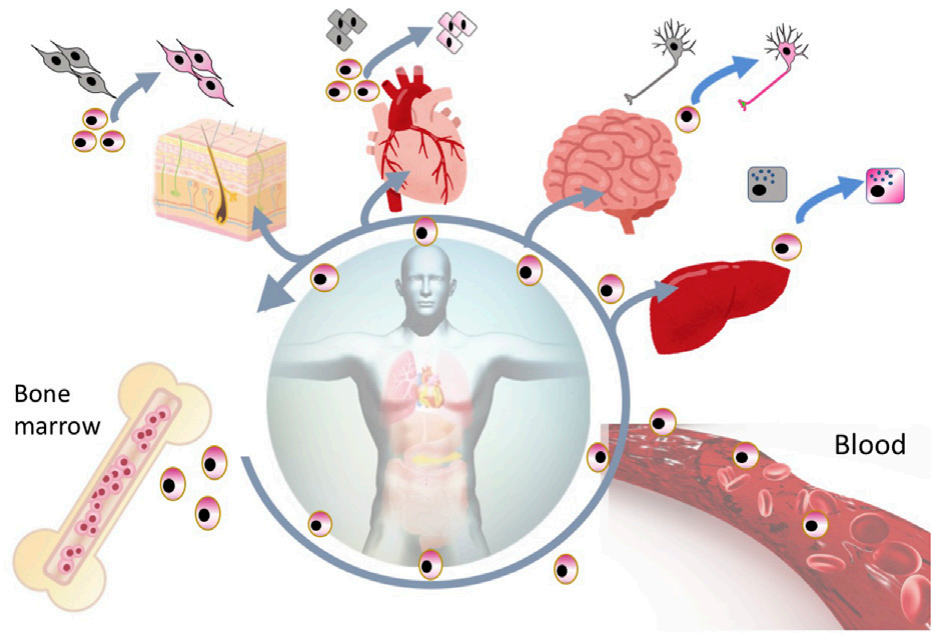
Reparative activity of endogenous Muse cells. Muse cells are considered constantly mobilized from the BM to the peripheral blood, and are supplied to every organ, where they replace minutely damaged/apoptotic cells by spontaneous differentiation into the damaged/apoptotic cell type.

SSEA-3 is the most generally used marker for identifying Muse cells from various tissues of multiple species, including human, mouse, rat, rabbit, swine, and goat (Chen et al., 2010; Guo et al., 2010; Kajitani et al., 2010; Squillaro et al., 2010; Yamauchi et al., 2010; Kuroda et al., 2010; Yang et al., 2013; Wakao et al., 2018; Yamada et al., 2018; Nitobe et al., 2019; Sun et al., 2020). In the BM, Muse cells comprise only 0.01%–0.03% of the total cell population, 1 in 1,000–3,000 mononucleated cells, and are shown to form loose clusters in humans (Kuroda et al., 2010). They are thought to be mobilized constantly from the BM to the peripheral blood, where they comprise 0.01%–0.2% of the mononuclear fraction (Figure 4) (Sato et al., 2020). The main source of peripheral blood-derived Muse cells is assumed to be the BM, while other organs such as the spleen can be candidate suppliers. Muse cells are then supplied to every organ through the bloodstream. Once in the organ, they locate sparsely throughout the organ’s connective tissue, as shown in the dermis, liver, spleen, pancreas, trachea, adipose tissue, dental pulp, and synovial tissue (Heneidi et al., 2013; Tsuchiyama et al., 2013; Kinoshita et al., 2015; Dezawa, 2016; Gimeno et al., 2017; Tian et al., 2017; Yamauchi et al., 2017; Nagano et al., 2019; Toyoda et al., 2019), as well as in the umbilical cord, an extra-embryonic tissue (Figures 3, 4) (Leng et al., 2019). Due to this wide distribution, Muse cells have not been linked to any histologic structure or particular niche-like structures (Table 1).

**TABLE 1:** Difference between muse cells vs. iPSCs vs. MSCs.

		Muse cells	MSCs	iPSCs
Source		Bone marrow organ connective tissues peripheral blood umbilical cord etc.	Bone marrow mesenchymal tissues umbilical cord	Artificially induced from fibroblasts blood cells other somatic cells
Marker expression	Surface markers	Positive for SSEA-3	Basically positive for CD73, CD90 and CD105 basically negative for CD11b, CD14, CD19, CD34, CD45 and CD79a	Positive for SSEA-3, SSEA-4, TRA-1-60, TRA-1-81
	Representative pluripotency associated genes	OCT3/4 (moderate), NANOG (moderate), SOX2 (moderate), KLF4 (moderate), LIN28 (low/no), TERT (low)	OCT3/4 (low/under detection; UD), NANOG (low/UD), SOX2 (low/UD), KLF4 (low/UD), LIN28 (low/UD), TERT (low/UD)	OCT4 (high), NANOG (high), SOX2 (high), KLF4 (moderate), LIN28 (high), TERT (high)
Applications		Cell therapy stem cell research	Cell therapy stem cell research	Stem cell reserch *in vitro* disease modeling drug development
Limitations		Do not proliferate unlimitedly	Differentiation ability do not proliferate unlimitedly	Tumorigenicity require HLA-matching for transplantation

Muse cells from the BM and organ connective tissue are double-positive for SSEA-3 and CD105, an MSC marker (Wakao et al., 2011), whereas peripheral blood-derived Muse cells are double-positive for SSEA-3 and CD45, a white blood cell marker (Sato et al., 2020). It is still unknown whether Muse cells switch classes when they are mobilized from the BM to peripheral blood or whether the two lines of Muse cells, SSEA-3(+)/CD105(+) and SSEA-3(+)/CD45(+), operate simultaneously (Sato et al., 2020).

Muse cells are also found in cultured MSCs and fibroblasts as a small fraction of the total cell population in multiple species, including human, mouse, rat, rabbit, swine, and goat (Kuroda et al., 2010; Wakao et al., 2011; Tsuchiyama et al., 2013; Yang et al., 2013; Ogura et al., 2014; Uchida et al., 2017b; Wakao et al., 2018; Yamada et al., 2018; Leng et al., 2019; Nagano et al., 2019; Sun et al., 2020; Iseki et al., 2021). Almost all MSCs and fibroblasts are positive for mesenchymal markers such as CD105. Muse cells contained in these cells are double positive for SSEA-3 and CD105, as mentioned above (Wakao et al., 2011). When MSCs and fibroblasts are separated into SSEA-3(+)/CD105(+) Muse cells and SSEA-3(-)/CD105(+) non-Muse MSCs/fibroblasts, their characteristics are different. Notably, SSEA-3(-)/CD105(+) non-Muse MSCs exhibit characteristics essentially identical to that of conventional MSCs, as seen by their expression of MSC markers such as CD105, CD90, and CD29, and their ability to differentiate into osteogenic, chondrogenic and adipogenic cells by cytokine induction (Wakao et al., 2011; Ogura et al., 2014). In the following text, SSEA-3(-)/CD105(+) non-Muse MSCs are called “non-Muse MSCs,” and MSCs that include several percent of Muse cells are called “MSCs.”

### Stress tolerance and high DNA damage-repairing capacity

Muse cells were initially discovered as a stress-tolerant subpopulation among MSCs that tolerate long-term incubation (∼16 h) in trypsin solution without nutrition. Later, they were shown to secrete factors involved in stress tolerance, including serpins and 14-3-3 proteins (Alessio et al., 2017). They were also shown to rapidly, within 6 h, activate homologous recombination and non-homologous end joining repair systems to repair DNA damages (Alessio et al., 2018). In addition, Acar et al. (2021) reported that the stress-activated protein kinase (SAPK)/Jun amino terminal kinase (JNK) signaling pathway plays an important role in stress tolerance of Muse cells. Due to these mechanisms, Muse cells are stress-tolerant and are able to survive in an environment under strong genotoxic stress (Alessio et al., 2018). Stress tolerance is essential for cells to play a role in controlling inflammation in damaged tissue, which is rich in cell debris and highly cytotoxic substances.

### Anti-inflammatory, anti-apoptotic, anti-fibrosis, and tissue-protection effects of Muse cells

The ability to produce cytokines and trophic factors relevant to the bystander effect is typically higher in Muse cells than in non-Muse MSCs/MSCs *in vitro*; therefore, Muse cells generally exhibit greater tissue-protective effects than non-Muse MSCs and MSCs *in vivo* (Kinoshita et al., 2015; Alessio et al., 2017; Yabuki et al., 2018; Yamada et al., 2018). The more potent bystander effect of Muse cells can be explained by their successful integration into the damaged target tissue and survival in the tissue as functional cells for an extended period of time, i.e., over 6 months, even in the case of allogenic cells without immunosuppressants (Yamada et al., 2018). Non-Muse MSCs and MSCs, on the other hand, do not efficiently reach damaged tissue but become trapped in the lung capillaries and disappear from the body within a couple of weeks. Thus, they do not remain in the damaged target tissue for an extended period of time like Muse cells (Yamada et al., 2018; Yamashita et al., 2020; Fujita et al., 2021; Shono et al., 2021).

**FIGURE 5:**
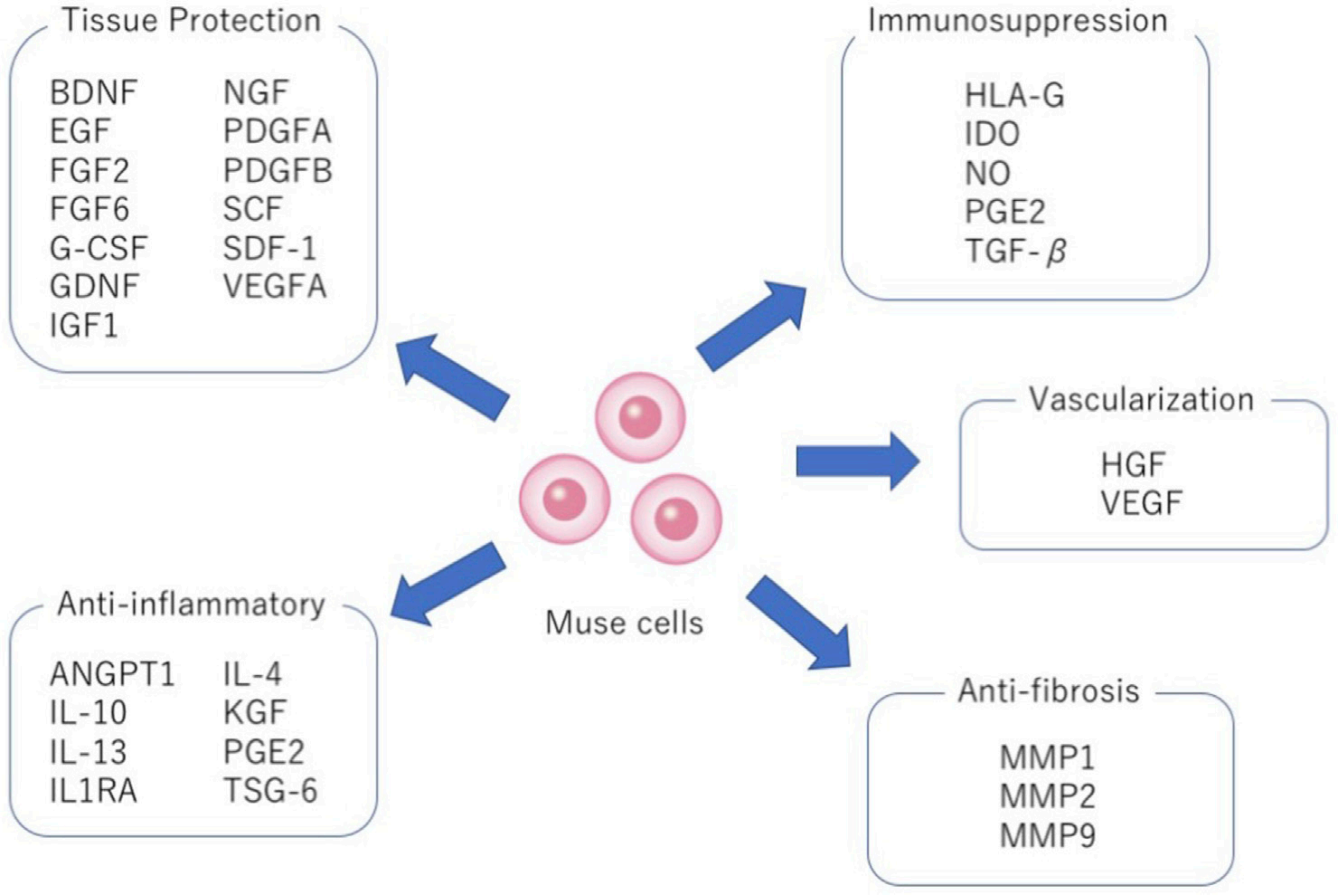
Pleiotropic effects of Muse cells. Production of factors relevant to tissue protection, anti-inflammation, immunosuppression, vascularization and anti-fibrosis by Muse cells.

As for anti-inflammatory effects, similarly like MSCs, Muse cells secrete various cytokines such as interleukin-4 (IL-4), IL-10, IL-13, transforming growth factor-β (TGF-β), and prostaglandin E2 (PGE2) (Figure 5). Among others, IL-10 and TGF-β, highly effective anti-inflammatory cytokines, are produced at higher levels in Muse cells than in MSCs. Granulocyte colony-stimulating factor (G-CSF) production was demonstrated to play a central role in protecting the blood-brain barrier and neural cells in Shiga toxin-producing Escherichia coli-associated encephalopathy (Ozuru et al., 2020). Similarly, other factors such as epidermal growth factor (EGF), KGF, VEGFA, PDGFA, PDGFB, FGF2, FGF6, ANGPT1, NGF, BDNF, GDNF, IGF1, IL1RA, SCF and SDF-1, cytokines relevant to anti-inflammatory, tissue protection, wound healing, and inhibition of apoptosis are produced at higher levels in Muse cells than in MSCs (Figure 5) (Kinoshita et al., 2015; Uchida et al., 2017b; Iseki et al., 2017; Yamauchi et al., 2017; Yabuki et al., 2018; Wang et al., 2019; Shono et al., 2021). Macrophages also significantly reduce the production of tumor necrosis factor-α (TNF-α) when co-cultured with Muse cells *in vitro.* Muse cells exhibited significant protective effects on the proliferation maintenance and intestinal barrier structure of intestinal epithelial crypt cells damaged by TNF-α through reduction of IL-6 and interferon-γ (IFN-γ) and increased release of TGF-β and IL-10 in the inflammation microenvironment (Sun et al., 2020).

Muse cells also produce matrix metalloproteases-1 (MMP1), MMP2, and MMP9. These MMPs are important for suppressing fibrosis because of their ability to degrade the extracellular matrix (Figure 5). In animal models of liver damage, chronic kidney disease, and acute myocardial infarction, intravenous injection of Muse cells delivered a statistically meaningful reduction of fibrosis compared with the MSCs or non-Muse MSCs groups (Uchida et al., 2017b; Iseki et al., 2017; Yamada et al., 2018).

Muse cells not only produce VEGF and HGF, known activators of neovascularization, but they also directly participate in neovascularization by spontaneously differentiating into vascular cells after homing to damaged tissues, as shown by animal models of acute myocardial infarction, chronic kidney disease, liver damage and aortic aneurism (Figure 5) (Uchida et al., 2017b; Hosoyama et al., 2018; Yamada et al., 2018; Shono et al., 2021). Muse cells were able to home to damaged tissues and spontaneously differentiate into CD31+ endothelial cells in the intimal layer and smooth muscle cells in the medial layer in a mouse aortic aneurism model (Hosoyama et al., 2018). Thus, Muse cells are efficient in vascular protection and neovascularization.

### Expression of genes relevant to pluripotency

The repertoire of genes relevant to pluripotency and the undifferentiated state expressed in Muse cells is similar to that in ES and iPS cells, but the expression level of those genes is generally lower in Muse cells than in ES and iPS cells. Due to this, Muse cells are characterized as “moderate pluripotent stem cells” (Wakao et al., 2011). The genes expressed include Oct3/4, Sox2, Nanog, Rex1, and PAR4, as well as bone morphogenic protein 4 (BMP4), deleted in azoospermia-like (DAZL), developmental pluripotency-associated protein (DPPA) 3 and 4, PR/SET domain 1 (Prdm1), SIX homeobox 4 (Six4), sprouty RTK signaling antagonist (SPRY) 1/2, and undifferentiated transcription factor 1 (UTF1) (Kuroda et al., 2010; Wakao et al., 2011; Alessio et al., 2017). In contrast to Muse cells, non-Muse MSCs and fibroblasts do not express pluripotency genes or express them at very low levels compared to those in Muse cells (Kuroda et al., 2010; Wakao et al., 2011). Consistently, the promoter regions of Nanog and Oct3/4 are less methylated in Muse cells than in non-Muse fibroblasts (Wakao et al., 2011).

Muse cells upregulate Oct3/4, Sox2, and Nanog gene expression levels 10 to 80 times higher in suspension than in adherent culture *in vitro* (Iseki et al., 2017). When comparing Muse cells directly collected from human peripheral blood with Muse cells collected from adherent cultured human BM-MSCs, peripheral blood-derived Muse cells had 30–90 times higher gene expression of Oct3/4, Sox2, and Nanog than BM-MSC-derived Muse cells (Sato et al., 2020). This suggests that pluripotency is highly enhanced in circulating Muse cells compared to tissue-resident Muse cells.

In the adherent state, those proteins were shown to be mainly located in the cytoplasm *via* immunocytochemistry. When transferred to suspension, Oct3/4, Sox2, and Nanog translocated to the nucleus (Wakao et al., 2018). Since Oct3/4, Sox2, and Nanog are transcription factors, they may not be fully activated in the adherent state due to being anchored to the cytoplasm but are activated in suspension by translocating to the nucleus (Wakao et al., 2018). Thus, the cellular state, suspension versus adherent, is one of the factors that control pluripotency gene expression level.

### Triploblastic differentiation ability of Muse cells

In single cell suspension culture, Muse cells start cell division and form clusters that are similar to ES cell-derived embryoid bodies (Kuroda et al., 2010; Wakao et al., 2011). Those single Muse cell-derived clusters exhibit positivity for the alkaline phosphatase reaction and express pluripotent markers such as Oct3/4, Nanog, Sox2, Rex1, and PAR4 at higher levels than in the adherent state, as mentioned above (Kuroda et al., 2010). After transferring the clusters to a gelatin-coated dish, cells adhere, expand from the cluster, and show spontaneous differentiation into cells positive for endodermal (GATA-6, cytokeratin 7, alpha-fetoprotein), mesodermal (GATA-4, Nkx2.5, smooth muscle actin, desmin), and ectodermal (MAP-2, neurofilament) markers, without any cytokine treatment. Thus, Muse cells are able to spontaneously generate triploblastic-lineage cells from a single cell (Kuroda et al., 2010). However, as the ratio of marker expression for ectodermal and endodermal lineages is several percent and that for mesodermal lineage is around 10%, the ratio of spontaneous differentiation is not high. Triploblastic differentiation ability is self-renewable at a single-cell level over generations, suggesting the pluripotent-like property of Muse cells (Kuroda et al., 2010; Ogura et al., 2014). Importantly, Muse cells collected directly from human bone marrow aspirate also exhibited the same ability (Kuroda et al., 2010). Therefore, the pluripotent-like property is not newly acquired by *in vitro* manipulation, nor are the cells modified under culture conditions, but it is suggested to be conferred on endogenous Muse cells.

Consistent with these observations, Muse cells secrete factors that may play a role in stem cell self-renewal, such as AKAP13, which anchors cAMP-dependent protein kinase and sustains proliferation, growth, and pluripotency, as well as molecules related to the LXR/RXR and FXR/RXR pathway and the cAMP-dependent protein kinase pathway, involved in self-renewal (Alessio et al., 2017).

Muse cells differentiate *in vitro* at a high rate (∼80%–95%) into various cell types, such as endodermal hepatic cells; ectodermal neural cells, keratinocytes, and melanocytes; and mesodermal adipocytes, osteocytes, renal-lineage cells, and cardiac cells, when specific sets of cytokines are adequately supplied (Wakao et al., 2011; Tsuchiyama et al., 2013; Amin et al., 2018). For example, human dermal-derived Muse cells were converted into melanin-producing functional melanocytes (Tsuchiyama et al., 2013; Yamauchi et al., 2017), human BM-Muse cells into ventricular cardiomyocytes (Amin et al., 2018), and mouse adipose-Muse cells into neuronal cells with physiological activity (Nitobe et al., 2019).

Muse cells are also able to differentiate into target cell types that belong to ectodermal, endodermal, and mesodermal lineages *in vivo*. Muse cells home to the damaged site by sensing S1P, produced by damaged cells, through S1P receptor 2 (S1PR2) (Yamada et al., 2018). After selective homing to the damaged site, they spontaneously differentiate into tissue-specific cells that comprise the tissue and replace damaged and dying cells, as shown by previous reports (Figure 2) (Uchida et al., 2017a, Uchida et al., 2017b; Iseki et al., 2017; Hosoyama et al., 2018; Yamada et al., 2018; Shono et al., 2021). The *in vivo* differentiation proceeds rapidly compared with *in vitro* cytokine-induced differentiation. Muse cells that homed to the post-infarct area of a stroke model started elongating neurites and expressing progenitor markers NeuroD and Mash1 within 3 days, forming a network-like structure, and expressed maturity markers MAP2 and NeuN at 7 days (Uchida et al., 2017a; Sato et al., 2020). They differentiated into neuronal (∼60% of homed Muse cells) and glial cells (15–25% of homed Muse cells), which integrated into the pyramidal and sensory tract. The integrated cells exhibited physiological functionality and delivered motor and sensory recovery (Uchida et al., 2015a). Differentiation into neuronal cells and subsequent functional recovery were also reported in models of perinatal hypoxic ischemic encephalopathy, brain hemorrhage, ALS, and Shiga toxin-producing Escherichia coli-related encephalopathy (Shimamura et al., 2017; Ozuru et al., 2020; Yamashita et al., 2020; Suzuki et al., 2021) In an acute myocardial infarction model, Muse cells homed to the post-infarct tissue, spontaneously differentiated into cells positive for troponin-I, sarcomeric actinin, and connexin 43 within 2 weeks, and exhibited calcium influx and efflux synchronous with heart activity (Yamada et al., 2018). In a liver damage model, homed Muse cells spontaneously differentiated into albumin(+) and Hep par1(+) hepatocytes, Lyve-1(+) sinusoid endothelial cells, cytokeratin-7(+) cholangiocytes, and CD68(+) Kupffer cells (Katagiri et al., 2016; Iseki et al., 2017; Shono et al., 2021). In addition to neuronal and glial cells, cardiac cells, and cells of the liver, Muse cells have been shown to differentiate *in vivo* into keratinocytes, hair follicular cells, dermal fibroblasts, vascular endothelial cells, smooth muscle cells, glomerular cells (podocytes, mesangial cells, and endothelial cells), and skeletal muscle cells (Kuroda et al., 2010; Kinoshita et al., 2015; Hosoyama et al., 2018; Sato et al., 2020; Fujita et al., 2021).

### Non-tumorigenicity

As Muse cells are endogenous to the living body, they are non-tumorigenic and may have a low risk of tumorigenesis. The expression level of genes relevant to the cell cycle and tumorigenicity is lower in Muse cells than in ES and iPS cells and is comparable to those in non-Muse fibroblasts (Wakao et al., 2011). Telomerase activity, an indicator of tumorigenic activity, is also low in Muse cells compared with iPS and HeLa cells and is more comparable to non-Muse fibroblasts (Wakao et al., 2011). The Lin28-let7 pathway is known to be important for maintaining pluripotency, and the higher expression of Lin28, known to act as an oncogene, in ES and iPS cells is relevant to tumorigenicity (Thornton and Gregory, 2012). Muse cells express the lower level of Lin28 due to the higher expression of let7 that antagonizes Lin28 (Simerman et al., 2016). This might be one of the mechanisms that are relevant to the non-tumorigenicity of Muse cells with maintaining pluripotency, similar to planarians (Simerman et al., 2016). Accordingly, human Muse cells transplanted into the testes of immunodeficient mice did not generate any tumors for up to 6 months (Kuroda et al., 2010; Ogura et al., 2014; Gimeno et al., 2017).

The doubling time of Muse cells, ∼1.3 days per cell division, is comparable to that of fibroblasts both in adherent and suspension cultures (Kuroda et al., 2010). They expand stably in adherent culture until they reach the Hayflick limit and can be grown on a clinically relevant scale (Kuroda et al., 2010; Wakao et al., 2018). As generally seen in somatic stem cells, Muse cells randomly repeat symmetric and asymmetric cell divisions during proliferation. Therefore, the purity of Muse cells after isolation gradually decreases by producing non-Muse cells as expansion proceeds, finally reaching a plateau of several percent, equal to the proportion of Muse cells in MSCs and fibroblasts (Wakao et al., 2018). Another interesting point is that, unlike ES and iPS cells which depend on LIF and BMP4 for stable proliferation, Muse cells depend more on FGF to maintain their proliferation and self-renewal abilities. The withdrawal of FGF substantially slows Muse cell proliferation and accelerates asymmetric cell division (Wakao et al., 2018).

### Muse cells are distinct from other somatic stem cells

Somatic stem cells are reported to reside in many tissues such as the BM, adipose tissue, dermis, and peripheral blood. The BM and PB contain, for example, HSCs, endothelial progenitors, MSCs, and VSEL stem cells (Caplan, 2008; Crisan et al., 2008; Ratajczak et al., 2008). In the dermis, cells such as skin-derived precursors, neural crest-derived stem cells, melanoblasts, perivascular cells, and endothelial progenitors are present (Nishimura et al., 2002; Fernandes et al., 2004; Murga et al., 2004; Middleton et al., 2005; Gimble et al., 2007; Crisan et al., 2008; Nagoshi et al., 2008; Biernaskie et al., 2009).

Muse cells are negative for c-kit/CD117 (a marker for HSCs and melanoblasts), CD34 (HSCs and endothelial progenitors), NG2 (perivascular cells), von Willebrand factor and CD31 (both endothelial progenitor markers), CD146 (perivascular cells), CD271 and Sox10 (neural crest-derived stem cells), Snai1 and Slug (skin-derived precursors), and Tyrp1 and Dct (melanoblasts). VSEL stem cells are reported to exist in the BM, human umbilical cord blood, and PB as CD34+/Lin-/CD45-nonhematopoietic cells, exhibiting a different marker expression profile than Muse cells. These collectively suggested that Muse cells are distinct from these stem/progenitor cells found in the BM, adipose tissue, dermis, and PB (Wakao et al., 2011).

## Muse cells as endogenous pluripotent-like reparative stem cells with anti-inflammatory effects

Muse cells have several advantages for therapeutic application:

• They can be delivered intravenously and do not require surgery for administration.
• Gene introduction and cytokine treatment are unnecessary since Muse cells spontaneously differentiate into tissue-constituent cells with few errors to replace damaged/apoptotic cells after homing into damaged tissues.
• Donor-derived Muse cells can be directly transferred to patients without HLA-matching or immunosuppressive therapy.
• Anti-inflammatory effects are long-lasting because Muse cells, both autologous and allogenic, survive in the host tissue as integrated cells for an extended period of time.
• Low risk of tumorigenesis since they are endogenous to the body and do not require gene manipulations to render pluripotency or to differentiate into target cell types.

Due to these unique characteristics, clinical trials for seven kinds of diseases, including acute myocardial infarction (Noda et al., 2020), stroke, and epidermolysis bullosa (Fujita et al., 2021), have all been conducted by intravenous drip of donor-derived Muse cells without HLA-matching or immunosuppressive treatment.

### Muse cells selectively home to sites of damage by intravenous injection

The main axis that controls the selective homing of endogenous and exogenously administered Muse cells to damage sites is the S1P-S1PR2 axis (Yamada et al., 2018). S1P is a sphingolipid produced from sphingosine, a component of the outer leaflet of the cell membrane, by converting enzymes sphingosine kinase 1 (SPHK1) and SPHK2 when cells are damaged, and is a pleiotropic lipid mediator involved in various cellular activities including migration, adhesion and inflammation in many types of cells, most notably in the immune and vascular systems (Obinata and Hla, 2019). Since the cell membrane exists throughout the cells of the body, S1P is one of the universal injury signals.

**FIGURE 6:**
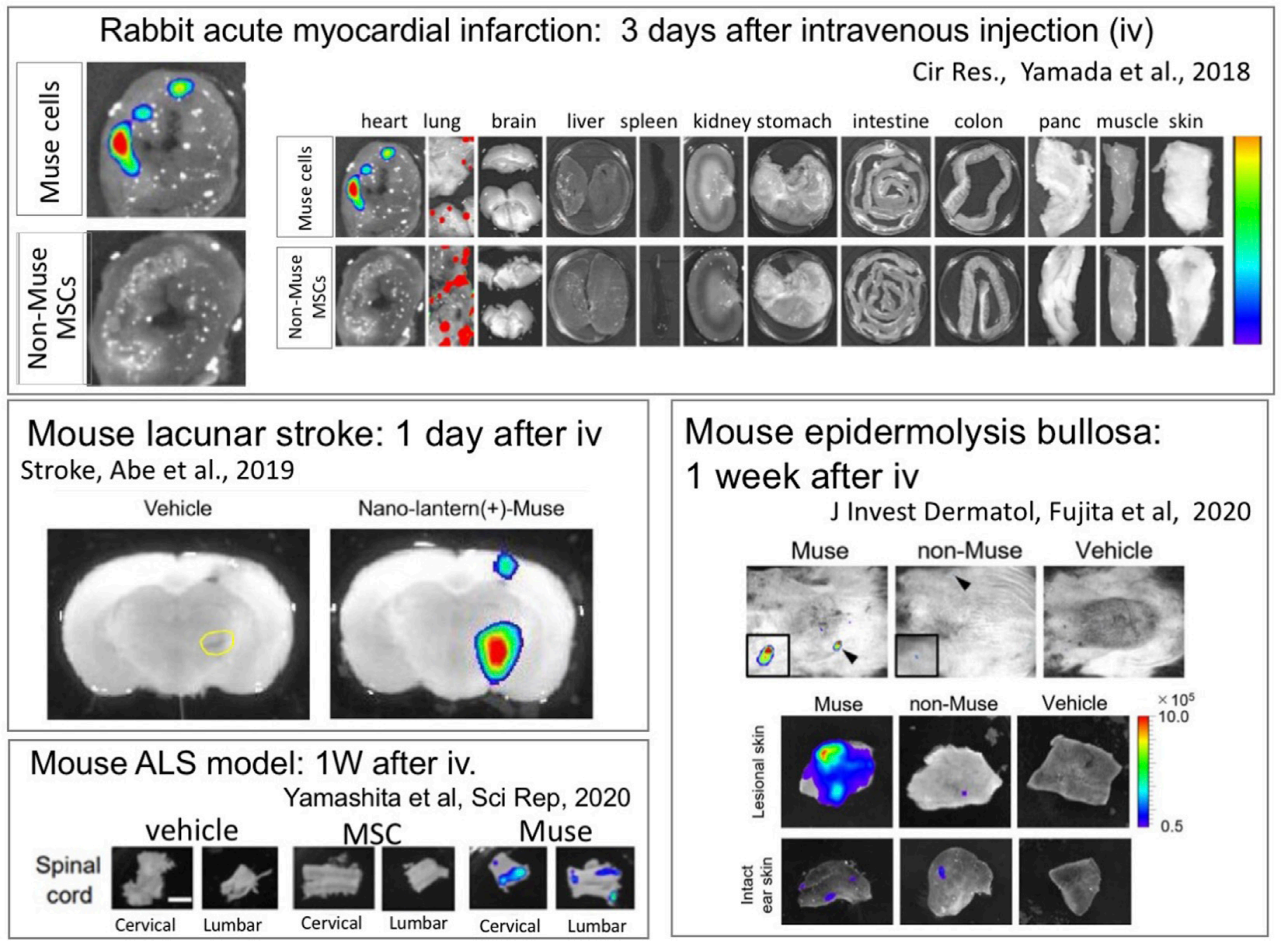
Intravenously administered Muse cells selectively home to the damaged site. Nano-lantern– or Akaluc-labeled human Muse cells were intravenously injected into a rabbit model of acute myocardial infarction (3 days after) (Yamada et al., 2018), a mouse models of lacunar infarction (1 day after) (Abe et al., 2020), ALS (1 week after) (Yamashita et al., 2020) and epidermolysis bullosa (1 week after) (Fujita et al., 2020), showing the selective homing of Muse cells into the damaged tissue.

There are five subtypes of S1P receptors, S1PR1∼S1PR5 (Obinata and Hla, 2019). S1PR1 promotes the egression of lymphocytes from thymus and secondary lymphoid organs, while S1PR2 retains their egression (Laidlaw et al., 2019; Obinata and Hla, 2019). Muse cells, on the other hand, mainly express S1PR2 that sense the location of damaged cells by sensing S1P and to migrate selectively to the damaged site (Figure 6) (Yamada et al., 2018). In a rabbit acute myocardial infarction, S1P concentration is substantially elevated in the border area of the infarct tissue 6 h after onset (Yamada et al., 2018). Similarly, in patients with acute myocardial infarction, the serum S1P level was quickly elevated at onset (Tanaka et al., 2018). The importance of the S1P-S1PR2 axis in Muse cells was confirmed by S1PR2 knockdown with small interference RNA in Muse cells and by co-administration of the S1PR2-specific antagonist JTE-013 in a rabbit acute myocardial infarction model. Both treatments largely impeded the specific homing of Muse cells to the post-infarct heart tissue. The S1PR2 agonist SID46371153, on the other hand, enhanced the migration of Muse cells towards the agonist *in vitro* (Yamada et al., 2018).

In contrast to Muse cells, intravenously injected MSCs mostly become trapped in the lung capillaries and do not home to damaged sites (Figure 6) (Yamada et al., 2018). In a rabbit acute myocardial infarction model, only a few or no intravenously injected MSCs integrated into the post-infarct tissue, whereas ∼14.5% of intravenously injected Muse cells were engrafted to the tissue (Yamada et al., 2018). Muse cells exhibited superiority over non-Muse MSCs and MSCs in selective homing to the sites of damage in several animal models, as shown by epidermolysis bullosa (Figure 6) (Fujita et al., 2020), ischemic stroke (Figure 6) (Uchida et al., 2017a), doxorubicin-induced nephropathy (Uchida et al., 2017b), ALS (Figure 6) (Yamashita et al., 2020), Shiga toxin-producing *Escherichia coli* (STEC)-associated encephalopathy(Ozuru et al., 2020), and perinatal hypoxic ischemic encephalopathy (Suzuki et al., 2021; Matsuyama et al., 2022).

### Muse cells survive in the tissue as tissue-constituent cells by *in vivo* differentiation

**FIGURE 7:**
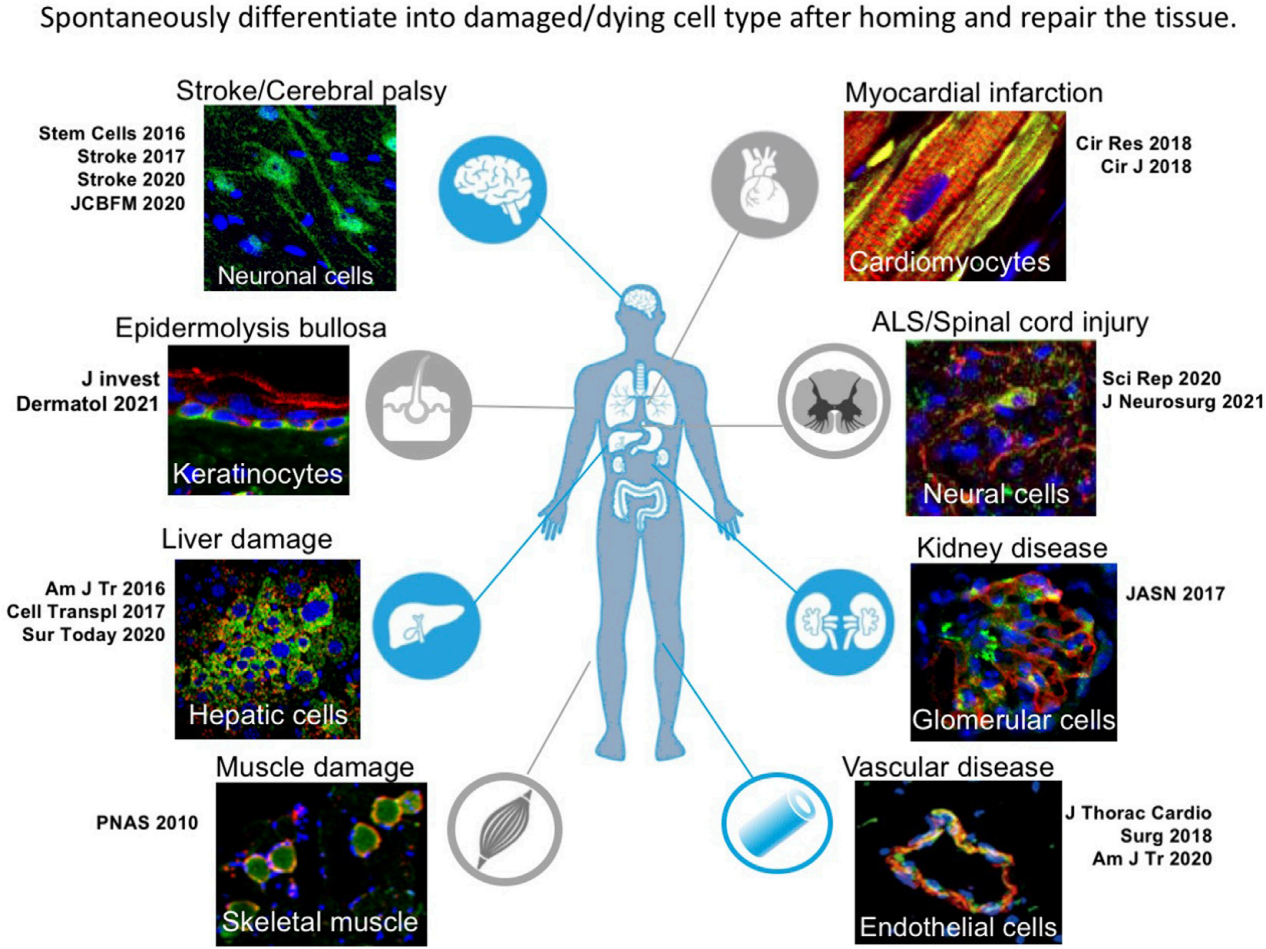
Tissue repair by spontaneous differentiation of Muse cells in each damaged tissue. Muse cells after homing to the damaged tissue spontaneously differentiate into cell types compatible to the tissue.

An outstanding characteristic of Muse cells as reparative stem cells is the ability to spontaneously and simultaneously differentiate into the damaged and apoptotic cell types that comprise the tissue after homing (Figure 7). This process proceeds rapidly compared with *in vitro* cytokine-induced differentiation. In most cases of cytokine induction into melanocytes, cardiomyocytes, osteocytes, adipocytes, neural, and hepatic lineage cells, it takes at least several months for ∼80% of Muse cells to differentiate into target cell types (Kuroda et al., 2010; Wakao et al., 2011; Tsuchiyama et al., 2013; Amin et al., 2018). *In vivo*, however, human Muse cells took ∼3 days to express early neural markers (Mash1 and NeuroD) and ∼7 days to express maturity markers (MAP2 and NeuN) in the post-infarct tissue of rat stroke model (Uchida et al., 2015b; Uchida et al., 2017a). Those human Muse cell-derived neuronal cells were shown to be incorporated into the pyramidal tract, including the pyramidal decussation, and into the sensory tract, as demonstrated by somatosensory-evoked potentials and the formation of synapses with host neuronal cells by 3 months, leading to statistically meaningful functional recovery (Uchida et al., 2015a). Neuronal functional recovery was also reported in perinatal hypoxic ischemic encephalopathy, brain hemorrhage, ALS, and Shiga toxin-producing Escherichia coli-related encephalopathy (Shimamura et al., 2017; Ozuru et al., 2020; Yamashita et al., 2020; Suzuki et al., 2021).

In the rabbit acute myocardial infarction model, intravenously injected rabbit and human Muse cells spontaneously differentiated into cardiac marker-positive cells (troponin-I, sarcomeric -actinin, and connexin 43) that exhibited calcium influx and efflux synchronous with heart activity recorded by an electrocardiogram (Yamada et al., 2018).

In mouse liver damage models, homed human Muse cells expressed liver progenitor markers (i.e., cytokeratin-19, DLK, OV6, alpha-fetoprotein) 2 days after intravenous injection and expressed mature hepatocyte markers (HepPar1, albumin, anti-trypsin) by 2 weeks, without fusing with host hepatocytes (Katagiri et al., 2016; Iseki et al., 2017). By 8 weeks, they expressed cytochrome P450, family 1, subfamily A, polypeptide 2 (CYP450 1A2) and glucose-6-phosphatase, enzymes related to drug metabolism and glycolysis, and delivered hepatic function recovery as seen in the increase of serum albumin level and decrease of total bilirubin levels, suggesting functional maturity of integrated Muse cells (Katagiri et al., 2016; Iseki et al., 2017). A similar result was obtained in a swine hepatectomy model with intravenous injection of allogenic Muse cells (Iseki et al., 2021).

Likewise, human Muse cells differentiated spontaneously into glomerular cells, WT-1(+)/podocin(+) podocytes, megsin(+) mesangial cells, and CD31(+)/von Willebrand factor(+) endothelial cells in a mouse chronic kidney disease model (Uchida et al., 2017b); dystrophin(+) skeletal muscle cells in a mouse muscle degeneration model (Kuroda et al., 2010); endothelial cells and smooth muscle cells in mouse aortic aneurism model (Hosoyama et al., 2018); and into keratinocytes, hair follicular cells, sweat gland cells, and capillary cells in a mouse epidermolysis bullosa model (Fujita et al., 2021).

Importantly, besides tissue-specific cell types, Muse cells directly participated in neovascularization by spontaneously differentiating into vascular cells, as shown in many tissue damage models (Katagiri et al., 2016; Uchida et al., 2017a; Hosoyama et al., 2018; Yamada et al., 2018; Fujita et al., 2021). Needless to say, neovascularization is a key point for tissue repair.

### Immune privilege of Muse cells

Immunologic rejection is a significant drawback of allogenic cell therapy (Zhao et al., 2019). When allogenic cells are infused or transplanted *in vivo* without proper HLA or major histocompatibility complex (MHC) matching or without using immunosuppressants, cells are quickly rejected by the host’s immune system (Ankrum et al., 2014). This reaction proceeds particularly quickly with xenogenic cell therapy; mouse cells transferred into rabbits were rapidly eliminated within 40 min, and rabbit cells transferred into swine were rejected within 2 h (Johnston et al., 1992; Kerr et al., 1999). In allogenic infusion or transplantation, rejection occurs under HLA/MHC mismatch; BM cells from BALB/c mice transplanted into C57BL/6 mice were rejected within 3 h in the earliest cases (Taylor et al., 2007). For these reasons, HLA/MHC matching should occur as often as possible. Furthermore, concomitant use of immunosuppressants is desirable, except in the cases of autologous or syngeneic infusion or transplantation.

Notably, allogenic Muse cells can survive for an extended period of time without HLA/MHC matching or immunosuppressant treatment; In a rabbit acute myocardial infarction model, intravenously injected allogenic Muse cells successfully homed into the post-infarct tissue, differentiated into cardiomyocytes, remained in the heart for 6 months but not in other organs, and maintained cardiac functional recovery at the same level as that observed in 2 weeks, even without immunosuppressants (Yamada et al., 2018). Xenogenic human Muse cells were shown to be survivable as differentiated functional cells in animal models without immunosuppressive therapy for a certain period of time, while shorter than that in allogenic Muse cells. Intravenously injected human Muse cells engrafted into damaged glomeruli without immunosuppressants survived as functional glomerular cells in the kidney for 5 weeks after administration, eventually being rejected by host immune cells by 7 weeks (Uchida et al., 2017a). Human Muse cells survived as physiologically active cardiomyocytes in the post-infarct heart tissue for 2 weeks after administration in the rabbit acute myocardial infarction model (Yamada et al., 2018) and survived in the bladder to ameriorate Hunner-type interstitital cystitis in a rat model for 4 weeks (Furuta et al., 2022), both without immunosuppressants. These models collectively suggest that Muse cells have a specific immune privilege mechanism that prevents their elimination by the host immune system.

Based on the facts above, clinical trials are all conducted by intravenous drip of allogenic Muse cells without HLA-matching and immunosuppressant treatment. One of the explainable mechanisms is the expression of HLA-G. Muse cells express HLA-A, -B, and -C (MHC class I) but not HLA-DR (MHC class II) on the cell surface when analyzed by FACS (Yamada et al., 2018). Notably, the majority of Muse cells (∼90%) express a special class of HLA molecules, HLA-G, at a substantially higher level than that in MSCs (∼5%) (Yamada et al., 2018). HLA-G is expressed in immune-privileged extravillous trophoblasts in the placenta, which can strongly suppress the immune response or inhibit the proliferation and maturation of maternal macrophages, T cells, B cells, NK cells, dendritic cells, and neutrophils (Dezawa, 2016). Therefore, HLA-G expression is suggested to protect Muse cells from host immunologic attack (Figure 5). However, HLA-G expression decreases as Muse cells differentiate after homing to the damaged tissue. Human Muse cells, which survived in the mouse glomeruli as differentiated podocytes, mesangial cells, and endothelial cells, lost HLA-G expression 2 weeks after infusion (unpublished data), suggesting the presence of an unknown mechanism allowing HLA-G-negative differentiated Muse cells to continue escaping from host immunological attack.

MSCs, widely reported as immunomodulatory cells, may give a hint for this. MSCs are useful for alleviating the symptoms of many autoimmune diseases such as GVHD, systemic lupus erythematosus, and multiple sclerosis (Le Blanc et al., 2004; Liang et al., 2010; Lublin et al., 2014). One of the mechanisms is the production of Indoleamine 2,3-dioxygenase (IDO), the major immunomodulatory protein (Fallarino et al., 2002; Meisel et al., 2004). IDO is known to inhibit the kynurenine pathway by promoting tryptophan degradation in T cells, thereby inhibiting T cell proliferation and inducing apoptosis. IDO is also involved in the maturation of regulatory T cells (Treg), which are essential for acquiring immune tolerance (Tipnis et al., 2010). Notably, Muse cells produce IDO at a similar level as MSCs (Figure 5) (Uchida et al., 2017a).

Besides IDO, many other proteins relevant to immunomodulation are secreted by MSCs; TGFβ1 and PGE 2 have been identified as important factors for maturing Tregs, suppressing proliferation and cytokine production of T cells and Natural Killer (NK) cells, and inhibiting the maturation of macrophages and dendritic cells, thus exerting a negative regulatory effect against immunological cells (Di Nicola et al., 2002; Aggarwal and Pittenger, 2005; Ryan et al., 2007; English et al., 2009; Nemeth et al., 2009; Spaggiari et al., 2009). HGF and nitric oxide (NO), both produced by MSCs, also suppress T cell proliferation (Ryan et al., 2007; Ren et al., 2008).

In addition to cytokine production, MSCs are reported to regulate the immune system through direct cell-cell contact. For example, Tipnis et al. (2010) reported that umbilical cord-derived MSCs promoted Treg maturation while inhibiting T cell proliferation, as well as dendritic cell differentiation and maturation by cell-cell contact. MSCs can induce T cell apoptosis *via* programmed cell death-1 (PD-1) and FASL (a ligand for FAS that induces apoptosis) (Augello et al., 2005; Akiyama et al., 2012). In this manner, MSCs use several strategies for immunomodulation. Interestingly, Muse cells produce IDO, as mentioned above, as well as TGFβ1, PGE2, and HGF, thus are expected to utilize the same mechanisms as MSCs (Figure 5) (Uchida et al., 2017b; Yabuki et al., 2018).

While immunoregulatory mechanisms in Muse cells are still not fully understood, the following mechanisms are proposed based on the above presented pieces of knowledge. At the early stage after intravenous injection, Muse cells may suppress T cells *via* expression of HLA-G to induce apoptosis of T cell that reacts against allogenic Muse cells, and *via* production of IDO to suppress T cell proliferation. When T cell apoptosis is induced, macrophages actively produce TGF-β, leading to Treg differentiation and maturation. If so, under the control of Tregs, T cell apoptosis may expand the region, locally or systemically. In parallel produced anti-inflammatory cytokines by Muse cells may synergistically contribute to the immune privilege of Muse cells.

### Clinical trials of Muse cells

The therapeutic effects and safety of CL 2020, a clinical-grade Muse cell product, were demonstrated in mouse models of epidermolysis bullosa and lacunar stroke (Abe et al., 2020; Fujita et al., 2020) (Table 2). Based on the safety and efficacy demonstrated in preclinical studies, seven trials related to various diseases and conditions, including acute myocardial infarction, stroke, epidermolysis bullosa, spinal cord injury, neonatal hypoxic-ischemic encephalopathy, ALS, and COVID-19 acute respiratory distress syndrome, are currently in progress utilizing intravenous infusion of donor-derived CL2020 (Table 3).

**TABLE 2:** Pre Clinical studies of muse cells.

Model of diseases	Animal species	Source of Muse cells	Administration method	Use of immunosuppressants	Effects	References
Diabetic skin ulcers	Mouse	Human adipose tissue	Local injection	No	Acceleration of wound healing	Stem Cells Transl. Med. (2015)
Stroke	Rat	Human bone marrow	Local injection	Yes	Replenishment of neurons and oligodendrocytes, reconstruction of neuronal circuit, improvements in motor and sensory functions,	Stem Cells (2015)
Ischemic stroke	Mouse	Human bone marrow	Local injection	No	Homing to ishemic area, recovery of motor function	PLoS One (2015)
Liver fibrosis	Mouse	Human bone marrow	Intravenous injection	No	Recovered total bilirubin and albumin attenuation of fibrosis, replenishment of hepatocytes,	Cell Transplant. (2017)
Intracerebral hemorrhage	Mouse	Human bone marrow	Local injection	No	Improvements in the water maze and motor function	Exp. brain Res. (2017)
Lacunar infarction	Mouse	Human bone marrow	Local injection	No	Replenishment of neuronal cells and oligodendrocytes, recovery of cylinder tests, loss of function by diphtheria toxin, safety assessment for 10 month	Stroke (2017)
Focal segmental glomerulosclerosis	Mouse	Human bone marrow	Intravenous injection	No	Replenishment of glomerular cells, improvement in urine protein, creatinine clearance, and plasma creatinine levels attenuated glomerular sclerosis and interstitial fibrosis	J. Am. Soc. Nephrol. (2017)
Aortic aneurysms	Mouse	Human bone marrow	Intravenous injection	No	Attenuation of aneurysmal dilation and size, tissue repair	J. Thorac. Cardiovasc. Surg. (2018)
Acute lung ischemia–reperfusion Injury	Rat	Human bone marrow	Injected into pulmonary artery	No	Recovery in alveolar-arterial oxygen gradient, left lung compliance and histological injury score	Cell Transplant. (2018)
Acute myocardial infarction	Rabbit	Rabbit bone marrow (autologous and allogenic) human bone marrow	Intravenous injection	No	Reduced infarct size, replenishment of cardiomyocytes, improvement of cardiac function	Circ. Res. (2018)
Epidermolysis bullosa	Mouse	Human bone marrow CL 2020 (clinical grade Muse cells)	Intravenous injection	No	Production of human collagen-7 and -17 in the mouse blisters, differentiation of human Muse cells into keratinocytes and other skin cells, reduction of ulcer, change of white hair to black hair	J Invest Dermatol (2020)
Lacunar stroke	Mouse	CL2020	Intravenous injection	No	Functional recovery in cylinder test in subacute-phase–treated and chronic-phase–treated animals	Stroke (2020)
Corneal scarring wound	Mouse tree shrew	Human abdominal lipoaspirate tissue	Placed with scaffold	Yes	Promoted tissue regeneration reduced scarring	Sci. Transl. Med. (2020)
*E. coli*-associated encephalopathy	Mouse	Human bone marrow	Intravenous injection	No	Suppressoin of inflammation, gliosis and edema, replenishment of neuronal cells, prolonged survival period	Mol. Ther. (2020)
Amyotrophic lateral sclerosis	Mouse	Human bone marrow	Intravenous injection	No	Improvement of scores in the rotarod, hanging-wire and muscle strength. Alleviated denervation and myofiber atrophy in lower limb muscles	Sci. Rep. (2020)
Spinal cord injury	Rat	Human bone marrow	Local injection	No	Improvement of motor function	Biochem. Biophys. Res. Commun. (2021)
Atopic dermatitis	Mouse	Human bone marrow	Subcutaneous injection	No	Alleviated scratching behavior, reduced dermatitis	Stem Cell Res Ther (2021)
Post-hepatectomy liver failure	Swine	Swine bone marrow (allogenic)	Intravenous injection	No	Improved hyperbilirubinemia, prothrombin international normalized ratio suppression of necrosis	Surg. Today (2021)
Thoracic spinal cord contusion injury	Rat	CL2020	Intravenous injection	Yes	Improvement of hindlimb motor function	J Neurosurg Spine (2021)
Extra-small partial liver transplantation	Rat	Human bone marrow	Intravenous injection	No	Vascular protection, maintenance of vascular flow proliferation of hepatocytes and liver sinusoidal endothelial cells	Am. J. Transplant (2021)
Perinatal hypoxic ischemic encephalopathy	Rat	Human bone marrow	Intravenous injection	No	Replenishment of neuronal cells and oligodendrocytes, improvements in motor and cognitive functions at 5 month	J. Cereb. Blood Flow Metab. (2021)
Severe acute pancreatitis	Mouse	Human bone marrow	Intravenous injection	No	Improvement of edema, suppressin of macrophage infiltration and necrosis, increased proliferating endogenous pancreatic progenitors	Surg Today (2022)
Hunner-type interstitial cystitis	Rat	Human bone marrow	Local injection	No	Attenuated increase of urinary frequency decrease of bladder capacity	Int Urogynecol J (2022)

**TABLE 3:** Clinical studies of muse cells.

Diseases	Phase	Clinical trial ID	Source of Muse cells	Administration method	Use of immunosuppressants	Effects	Adverse effects	Pre-clinical model	References
ST-elevation myocardial infarction	Ⅰ/Ⅱ	JapicCTI-183834	CL2020	Intravenous administration	No	Increased left ventricular filling dynamics decreased wall motion score index	All events were determined to be unrelated to the CL2020 during the trial	Circ. Res. (2018)	Circ. J. (2020)
	Ⅱ/Ⅲ	JapicCTI-195067	CL2020	Intravenous administration	No	On going	On going	Circ. Res. (2018)	
Cerebral infarction	Ⅰ/Ⅱ	JapicCTI-184103	CL2020	Intravenous administration	No	On going	On going	Stem Cells (2015) PLoS One (2015)	
Epidermolysis bullosa	Ⅰ/Ⅱ	JapicCTI-184563	CL2020	Intravenous administration	No	Decreased the total size of the ulcers	One patient showed paraesthesia within 24 h after infusion and resolved in 14 days, which was considered a possible CL2020-related side-effect	J Invest Dermatol (2020)	J. Eur. Acad. Dermatol. Venereol. (2021)
Spinal cord injury	Ⅰ/Ⅱ	JapicCTI-194841	CL2020	Intravenous administration	No	On going	On going	J Neurosurg Spine (2021)	
Amyotrophic lateral sclerosis (ALS)	Ⅰ/Ⅱ	jRCT2063200047	CL2020	Intravenous administration	No	On going	On going	Sci. Rep. (2020)	
Acute respiratory distress syndrome induced by COVID-19	Ⅰ/Ⅱ	jRCT2043210005	CL2020	Intravenous administration	No	On going	On going		
Neonatal hypoxic ischemic encephalopathy	Ⅰ/Ⅱ	NCT04261335	CL2020	Intravenous administration	No	On going	On going		BMJ open (2022)

In the first-in-human trial of Muse cells in acute myocardial infarction, 1.5 × 10^7 CL2020 cells (2.1 ± 0.1 × 10^5 cells/kg) were intravenously infused at 4.1 ± 1.0 days after onset. No adverse events that would prevent the advancement of the clinical study were observed throughout the clinical study period. The function of the heart, indicated by the left ventricular ejection fraction and wall motion index score, significantly improved from ∼40% to ∼52% at 12 weeks after CL2020 treatment (Noda et al., 2020). A phase 1/2 open-label study for adult epidermolysis bullosa was also recently published. Five patients received a single injection of CL2020 cells, which reduced the ulcer size significantly for up to 3 months with statistical significance (Fujita et al., 2021). A randomized, double-blind, placebo-controlled clinical trial was conducted to evaluate the safety and efficacy of a single dose CL2020 treatment within 14–28 days after the onset (subacute phase) of cerebral infarction in patients who continued to exhibit physical dysfunction after standard acute treatment. The patients were followed for up to 52 weeks, confirming the safety and efficacy of CL2020. The highlights of the conference presentation are outlined in https://www.lsii.co.jp/assets/pdf/en/20210518-1.pdf.

### Anti-inflammatory effect of Muse cells *in vivo*

Administration of Muse cells significantly suppressed the infiltration of inflammatory cells such as macrophages and neutrophils and effectively alleviated edema at the site of injury in mouse aortic aneurysm, porcine post-hepatectomy liver failure (PHLF), rat interstitial cystitis, and a severe pancreatitis model (Hosoyama et al., 2018; Iseki et al., 2021; Fukase et al., 2022; Furuta et al., 2022).

In the rat ischemia-reperfusion lung injury model, intravenously injected human Muse cells delivered better recovery in the arterial oxygen partial pressure to fractional inspired oxygen ratio, alveolar-arterial oxygen gradient, lung compliance, and edema on day three and day five. Human Muse cells homed more efficiently to the injured lung than human MSCs. In the injured lung, Muse cells suppressed apoptosis and stimulated the proliferation of host alveolar cells by producing beneficial substances such as KGF, HGF, ANGPT1, and PGE2 (Yabuki et al., 2018).

G-CSF production was recently demonstrated to play a central role in Muse cells to protect neurons and the blood-brain barrier (BBB) in Shiga toxin-producing Escherichia coli-associated encephalopathy, a condition in which verotoxins cause severe inflammation, destroy the BBB and induce brain edema and extensive apoptosis, finally converging onto brain tissue damage and gliosis (Ozuru et al., 2020). In this case, intravenous injection of human Muse cells selectively homed into the mouse brain and spinal cord that were being attacked by verotoxins and delivered anti-inflammatory effects in the acute phase mainly by G-CSF production (Ozuru et al., 2020). Muse cells that homed to the brain and spinal cord provided BBB protection, significant amelioration of edema, suppression of apoptosis, and replacement of damaged neuronal cells by spontaneous differentiation, leading to a higher survival rate of infected animals (Ozuru et al., 2020).

HGF and VEGF that protect against tissue damage are also known to promote general tissue repair (Uchida et al., 2017b; Iseki et al., 2017). HGF and VEGF, which are more highly expressed in Muse cells than MSCs, had a statistically meaningful protective effect on liver sinusoidal endothelial cells and exhibited less necrosis in Muse cell-treated rat and swine liver damage models (Uchida et al., 2017b; Iseki et al., 2017; Iseki et al., 2021; Shono et al., 2021). When the production of HGF and VEGF was suppressed by siRNA in Muse cells, these protective effects became largely impaired (Shono et al., 2021).

In the damaged brain tissue of neonatal hypoxic-ischemic encephalopathy, Muse cells ameliorated the effects of excitotoxic brain glutamatergic metabolites and suppressed microglial activation, as shown by magnetic resonance spectroscopy and positron tomography, respectively (Suzuki et al., 2021).

In animal models of PHLF, renal injury, pancreatitis, and atopic dermatitis, Muse cells reduced the production of inflammatory cytokines (IL-1B, IL-6, IL-17A, IL-33, TNF) and increased the expression of anti-inflammatory cytokines (IL-10, PGE2, TGF-β, TSG-6) and growth factors (IGF1, VEGFA, VEGFB, VEGFC, VEGFD, EGF, TGFA), contributing to tissue protection and regeneration in the injured organs rather than MSCs (Uchida et al., 2017b; Hosoyama et al., 2018; Fei et al., 2021; Iseki et al., 2021; Fukase et al., 2022; Furuta et al., 2022).

The anti-inflammatory and tissue-protective effects of Muse cells have also been confirmed in human clinical trials. The administration of CL2020 to patients with epidermolysis bullosa reduced skin ulcer pain and decreased the liver inflammatory markers AST and ALT in chronic inflammation-induced liver injury (Fujita et al., 2021). Thus, Muse cells could be an effective treatment for diseases related to inflammation.

Uncontrolled inflammation causes fibrosis in the chronic phase (Giannandrea and Parks, 2014). Muse cells produce matrix metalloproteases like MMP1, MMP2, and MMP9, and notably, MMP9 is produced by Muse cells, not by non-Muse MSCs (Iseki et al., 2017). MMPs are important for suppressing fibrosis because they degrade an excessive amount of extracellular matrix (Giannandrea and Parks, 2014). In animal models of liver damage, chronic kidney disease, and acute myocardial infarction, intravenously injected Muse cells exhibited a statistically meaningful reduction of fibrosis compared with the MSCs or non-Muse MSCs (Uchida et al., 2017a; Iseki et al., 2017; Yamada et al., 2018; Iseki et al., 2021). Since the anti-fibrotic effect is mainly required after the acute phase, MSCs and non-Muse MSCs showed limited effectiveness compared to Muse cells since MSCs and non-Muse MSCs do not home to damaged tissue, nor do they remain in the tissue or the body for more than 2 weeks after administration (Uchida et al., 2017b; Yamada et al., 2018; Fei et al., 2021; Iseki et al., 2021).

## Future perspectives

Conventional MSCs are currently applied in clinical studies. Nevertheless, they give rise to confined cell types, namely chondrocyte, osteocyte, or adipocyte lineages (Pittenger et al., 1999), and their therapeutic effects are largely explained by trophic effects rather than cell replacement, unlike Muse cells (Fu et al., 2017). By contrast, Muse cells possess spontaneous triploblastic differentiation ability *in vivo* and can specifically home to damaged tissues *via* the S1P-S1PR2 axis. Thus, after simple intravenous administration, Muse cells can selectively home to the damaged site and repair the damaged tissue by spontaneous non-fusion-based differentiation to replace damaged and apoptotic tissue-component cells (Kushida et al., 2018). Therefore, Muse cell-based therapies do not need a surgical operation to deliver the cells to the target tissue and are easily accessible. In addition, Muse cells possess stronger DNA repair ability than MSCs (Alessio et al., 2018). The stronger DNA repair ability of Muse cells suggests a lower risk of tumorigenesis and mutations. Furthermore, because Muse cells are immune-privileged, they can be used without HLA matching or immunosuppressive therapy (Yamada et al., 2018). The pluripotency of Muse cells combined with their efficient reparative function and immunomodulatory properties indicate that these cells may be a more realistic choice than MSCs for next-generation cell therapy for treating inflammatory and tissue destructive diseases.
